# An Effective Correction Method for Seriously Oblique Remote Sensing Images Based on Multi-View Simulation and a Piecewise Model

**DOI:** 10.3390/s16101725

**Published:** 2016-10-18

**Authors:** Chunyuan Wang, Xiang Liu, Xiaoli Zhao, Yongqi Wang

**Affiliations:** 1 School of Electronicand Electrical Engineering, Shanghai University of Engineering Science, Longteng Road No. 333, Shanghai 201620, China; xiangliu09@fudan.edu.cn (X.L.); zhaoxiaoli@sues.edu.cn (X.Z.); wyq17008@sues.edu.cn (Y.W.); 2 School of Computer Science, Shanghai Key Laboratory of Intelligent Information Processing, Fudan University, Shanghai 200433, China

**Keywords:** sensor correction, feature points detection, multi-view simulation, visual difference compensation, piecewise correction

## Abstract

Conventional correction approaches are unsuitable for effectively correcting remote sensing images acquired in the seriously oblique condition which has severe distortions and resolution disparity. Considering that the extraction of control points (CPs) and the parameter estimation of the correction model play important roles in correction accuracy, this paper introduces an effective correction method for large angle (LA) images. Firstly, a new CP extraction algorithm is proposed based on multi-view simulation (MVS) to ensure the effective matching of CP pairs between the reference image and the LA image. Then, a new piecewise correction algorithm is advanced with the optimized CPs, where a concept of distribution measurement (DM) is introduced to quantify the CPs distribution. The whole image is partitioned into contiguous subparts which are corrected by different correction formulae to guarantee the accuracy of each subpart. The extensive experimental results demonstrate that the proposed method significantly outperforms conventional approaches.

## 1. Introduction

Multi-angle remote sensing, providing multi-angle observations of the same scene, enhances human’s ability to monitor and identify the Earth’s surface [1]. However, to successful use multi-angle images, the distortions caused by imaging from multiple viewing angles must be removed [2]. Geometric correction, especially in the case of imaging with large viewing angles, is an indispensable preprocessing step for the processing and applications of remote sensing images [3]. There are two key factors that can affect correction accuracy. One is how to effectively detect and accurately match the feature points between the reference image and the distorted image. The other is how to precisely estimate the model parameters using control points (CPs, i.e., matched feature points).

Previous studies of feature point extraction methods can be divided into three categories. The first category is based on the image gray that uses the gray differences around a pixel of an image [4]. The second category is based on the boundary. Detecting objects include invariant corners at the maximum curvature of the boundary chain code and intersection points after polygon approximation [5]. The third category is based on the parameter model that models each pixel of an image [6]. Since the same target in the distorted image often exhibits local distortions and gray differences compared to in the reference image, the second category method that depends on the edge quality, and the third category method that is restricted to the parametric model are unsuitable. The first category method is comparatively simple and easy to combine with other methods. According to the evaluation of various feature point extraction algorithms in [7], Scale-invariant feature transform (SIFT) [8] can provide high stability of image translation, rotation, and scaling. However, its modeling principle does not include complete affine invariance, which is only robust to a certain degree of angle changes in practice. Afterwards, Morel [9] achieved an increasing number of extracted points by simulating scale and parameters related to the optical axis of the imaging sensor and normalizing parameters related to translation and rotation. However, the algorithm is more complex to perform the simulation and normalization for each image pairs, and it is time consuming in practice. In this paper, a new CPs extraction algorithm is proposed by compensating the perspective difference for large angle (LA) images. A reference feature point set is formed by simulating the multi-views of the reference image and performing feature point detection on simulated images. The proposed algorithm ensures effective matching of feature points between the reference image and LA images.

Moreover, how to precisely estimate model parameters is another important aspect for correction accuracy [10,11]. Goncalves [12] experimentally proved the influence of the CP distribution on solving model parameters. The correction accuracy achieved by a uniform distribution of CPs is better than it obtained with a non-uniform set [13]. In addition, traditional methods usually employ the same correction model for a whole image [14] while few have considered the serious local distortions and resolution changes causing by LA imaging. Also, the large dimensions of an image would obviously magnify the error correction model parameters. Assuming that a 1000 × 1000 image is processed using the second-order polynomial model, the order of magnitude will reach 106. The piecewise linear functions [15,16,17], which divide the images into triangular elements by Delaunay’s triangulation method, and take affine transformation as the mapping function, are suitable to reduce local geometric distortions. However, the correction accuracy possible using the affine transformation model is lower than that of using a projection transformation model for LA images. The projection transformation model additionally requires at least five CP pairs. Consequently, the proposed piecewise correction algorithm is indispensable for LA image correction. Firstly, the image is separated into grids according to the variability of spatial resolution. Severely distorted are as have relatively intensive grid distributions. Based on these grids, the overall CP distribution is controlled and the piecewise location is determined. Then, CPs are optimized by joining the CPs distribution measurement (DM) to select the representative CPs in each grid. Finally, subparts deduced from the piecewise location are corrected by different correction formulae using the optimized CPs.

The rest of this paper is organized as follows: In Section 2, CP extraction based on multi-view simulation (MVS) is introduced. The piecewise correction algorithm is advanced with the optimized CPs by DM are described in Section 3. In Section 4, experimental results are presented to quantify the effectiveness of the new method. The conclusions of this paper with a summary of our results are provided in the final section.

## 2. CPs Extraction Based on Multi-View Simulation

**Figure 1:**
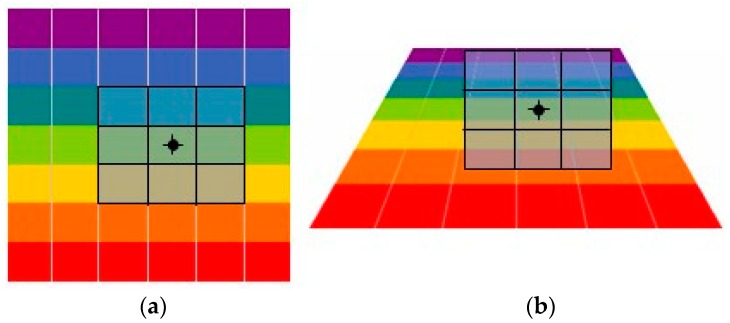
The changes of neighborhood pixels (**a**) the reference image; (**b**) the large angle image.

**Figure 2:**
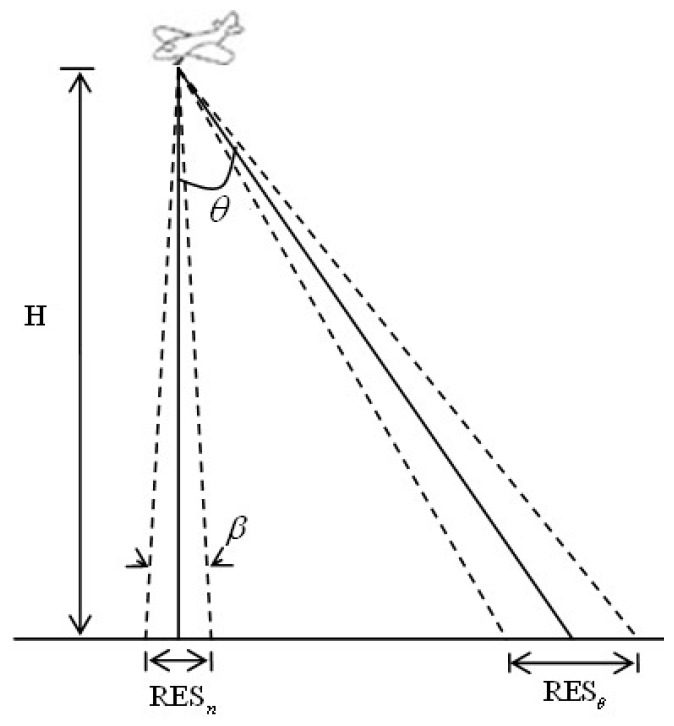
Spatial resolution of off-nadir and nadir.

SIFT, having excellent performance against scaling, rotation, noise and illumination changes, has been applied widely in the feature detection field. However, the modeling process does not consider the imaging oblique angle which results in the change of neighborhood pixels as shown in Figure 1. For example, in the process of the scale space extremum detection, determining whether the current pixel is a maximum value or minimum value requires 26 pixels in the neighborhood as a reference. If the reference pixels are changed, such as resolution reduction and gray distortion caused by LA imaging, the result of the extreme value would be changed. As another example, the process of the key point description also uses the gradient and direction of neighboring pixels as a reference. If the reference pixels were changed, the description vector would be changed which can reduce the matching probability. In the [18], the impact of LA imaging on spatial resolution is analyzed. For LA images, along the seriously oblique direction, the spatial resolution is decreasing with the increasing of imaging angles as shown in Figure 2. The off-nadir ground resolution $RES_{\theta }$ is given by:

(1) $$RES_{\theta }=\frac{\beta H}{\cos^{2}\theta }=\frac{RES_{n}}{\cos^{2}\theta }$$

where $RES_{n}$ is the nadir’s resolution, *θ* is the angle from nadir. The off-nadir resolution is reduced by a factor of cosine squared of *θ*. Equation (1) demonstrates that the resolution compression is nonlinear when the imaging angle is larger than 45°. The performance of SIFT is stable within 50° while it is obviously ineffective beyond 50°, which is shown in the following experimental Section 4.1. The experimental results are consistent with the theoretical analysis.

Based on the above analysis, in order to compensate viewing angle differences between the reference image and LA images, SIFT is extended to the multi-views space in this paper. The transformation model employed in MVS is described in Section 2.1. The detailed procedure of CPs extraction based on MVS is introduced in Section 2.2.

### 2.1. Multi-View Transformation Model

The projection relation of ground scene *I*_0_ to digital image *I* can be described as:

(2) $$I=ATI_{0}$$

where ***A*** and ***T*** are projection and translation, respectively.

According to the affine approximation theory [19], the above model can be expressed as:

(3) $$I(x,y)\to I(ax+by+e_{1},cx+dy+e_{2})$$

If any determinant of ***A*** is strictly positive, following the Singular Value Decomposition (SVD) principle, ***A*** has a unique decomposition:

(4) $$\begin{array}{ll} A & =\left[\begin{array}{cc} a & b\\ c & d \end{array}\right]=H_{\lambda }R_{1}(\psi )tR_{2}(\phi )\\ & =\lambda \left[\begin{array}{cc} \cos\psi & -\sin\psi \\ \sin\psi & \cos\psi \end{array}\right]\left[\begin{array}{cc} t & 0\\ 0 & 1 \end{array}\right]\left[\begin{array}{cc} \cos\phi & -\sin\phi \\ \sin\phi & \cos\phi \end{array}\right] \end{array}$$

where λ > 0, λ is a zoom parameter, ***R***_1_ and ***R***_2_ are two rotation matrices, and *t* expresses a tilt, namely a diagonal matrix with first eigenvalue *t* > 1 and the second eigenvalue equal to 1.

**Figure 3:**
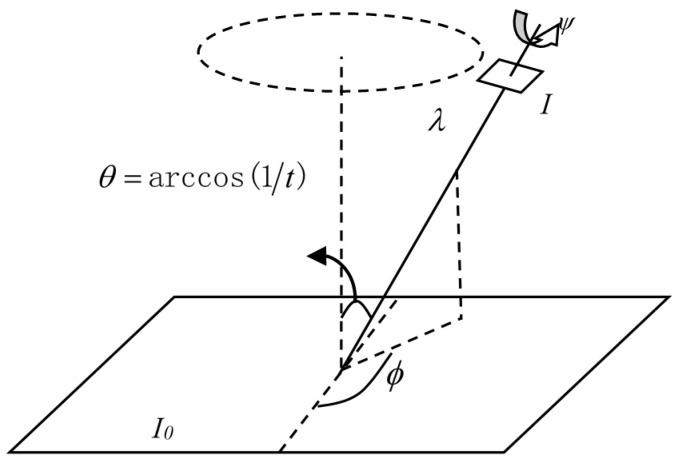
Geometric explanation for the matrix decomposition of transformation.

The geometric explanation for the matrix decomposition of transformation is shown in Figure 3. The angle *φ* is called longitude, which is formed by the plane containing the normal and the optical axis with a fixed vertical plane, where *φ* = ∊[0,*π*). The angle *θ* = arccos(1/*t*) is called latitude, which is formed by the optical axis with the normal to the image plane *I*_0_, where *θ* = ∊[0,*π*/2). *ψ* is the rotation angle of the imaging sensor around the optical axis. As seen in Figure 3, the motion of the imaging sensor leads to a parameter change from the positive perspective (*λ*_0_ = 1, *t*_0_ = 1, *φ*_0_ = *ψ*_0_ = 0) to the oblique angle (*λ*, *t*, *φ*, *ψ*), which represents the transformation *I*(*x,y*) →*I*(*A*(*x,y*)), where the longitude *φ* and the latitude *θ* determine the amount of perspective changes. The MVS is mainly based on two factors: the longitude *φ* and the latitude *θ*. *θ* is represented by the tilt parameter *t* = |1/cosθ| which measures the oblique amount between the reference image and LA images. The oblique distortion causes a directional subsampling in the direction given by the longitude *φ*.

The Affine SIFT (ASIFT) approach [9] operates on each image to simulate all distortions caused by a variation of the camera optical axis direction, and then it applies the SIFT method. ASIFT provides robust image matching between the two images due to the viewpoint change. However, ASIFT is time consuming. Assuming that the tilt and angle ranges are [*t_min_*, *t_max_*] = [1,4$\sqrt{2}$] and [*φ*_min_, *φ*_max_] = [0°, 180°], and the sampling steps are Δ*t* = $\sqrt{2},$ Δ*φ* = 72°/*t*. The complexity of the ASIFT approach is 1 + (|*Γ_t_*|−1)(180°/72°) = 1 + 5 × 2.5 = 13.5 times as much as that of a single SIFT routine, where $\left|\Gamma_{t}\right|=\left|\left\{1,\sqrt{2},2,2\sqrt{2},4,4\sqrt{2}\right\}\right|=6$. Even if the two-resolution scheme is applied, the ASIFT complexity is still more than twice that of SIFT. On the other hand, multiple matching of ASIFT results in matching point redundancy which demands huge calculating quantity and long computing time during the CPs optimization process. Even so, ASIFT provides a good idea for extracting CPs between the two images of viewing angle differences.

**Figure 4:**
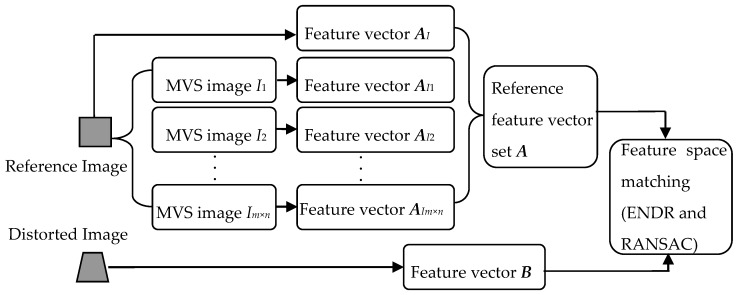
The overview of the proposed algorithm based on MVS.

Inspired by ASIFT, the new CPs extraction algorithm is proposed by simulating multi-views of the reference image to compensate the viewing angle difference for LA images. And then the new algorithm applies the SIFT to simulated images to construct a reference feature points database. Finally, feature matching is conducted between the reference feature point database and the feature points set detected from the LA image. The overview of the proposed algorithm is shown as Figure 4.

### 2.2. Procedure of CPs Extraction Based on Multi-View Simulation

The proposed algorithm proceeds by the following steps:

1. The reference image *I* is transformed by simulating all possible affine distortions by changing the camera optical axis orientation. In this process, the oblique transform is simulated by the tilt parameter *t*. Assuming that the *y* direction is the oblique direction, it represents *I*(*x,y*)→*I*((*x,ty*), which is equivalent to perform a *y* directional *t*-subsampling. Therefore, an antialiasing filter is previously applied, namely the convolution by a Gaussian with standard deviation $c\sqrt{t^{2}-1}.$ This ensures a very small aliasing error.
2. The simulated images of the reference image are calculated by the tilt parameter *t* and the longitude *φ* with sampling step lengths. When the latitude *θ* increases at a fixed degree, the image distortion is growing. Hence, the sampling density of *θ* should increase with the increasing of *θ*. As a result, the corresponding tilt parameter *t* follows a geometric series *t_k_*_1_ = 1,*u*,*u*^2^,…,*u^m^*(*k*_1_ = 0,1,…,*m*), with *u* > 1. Meanwhile, the image distortion is more drastic by a fixed longitude displacement Δ*φ* at the larger latitude *θ* = arccos(1/*t*). Hence, the longitudes *φ* are an arithmetic series for each tilt $\phi_{k2}=0,v/t,\ldots ,nv/t$ (*k*_2_ = 0,1,…,*n*), where the integer *k*_2_ satisfies the condition $(k_{2}v/t$ < 180°). The calculation of simulated images can be described as:
  (5) $$I_{k}(t_{k1},\phi_{k2})=\left[\begin{array}{cc} \cos\phi_{k2} & -\sin\phi_{k2}\\ \sin\phi_{k2} & \cos\phi_{k2} \end{array}\right]\left[\begin{array}{cc} t_{k1} & 0\\ 0 & 1 \end{array}\right]I\;\;\;\left(k=1,...,m\times n\right)$$
  where the ***I****_k_* expresses a set of simulated images of the reference image *I*, namely *I*_1_, *I*_2_,*…*,*I_m_*_×*n*_.
3. The feature point detection and description (SIFT) are applied to the reference image *I* and the simulated images *I*_1_, *I*_2_,*…*,*I_m_*_×*n*_. The feature vector sets ***A****_I_*,***A****_I_*_1_,***A****_I_*_2_,…,***A****_Im_*_×*n*_ are established which are combined together to form the reference feature vector set ***A***:
  ***A*** = ***A****_I_*∪***A****_I_*_1_∪***A****_I_*_2_∪…∪***A****_Im_*_×*n*_ (6)
4. The feature vector set ***B***, obtained from the distorted LA image, is matched with the reference feature vector set ***A*** by employing the European neighbor distance ratio criterion (ENDR) [20] and Random Sample Consensus (RANSAC) [21].

The two-resolution scheme [9] is applied to the proposed algorithm to reduce the computation complexity. With this, matched feature points, namely CPs, are extracted.

## 3. Piecewise Correction with Optimized CPs Based on Distribution Measurement

For different correction models, the required minimum number of CPs is different. However, a larger number of CPs doesn’t achieve a better root mean square error (RMSE) when CPs are concentrated in a local area. The CPs which are uniformly distributed in the image can achieve accurate model parameter estimation. To quantify the uniformity of CPs, the concept of DM is introduced based on information entropy. Then the proposed piecewise correction algorithm with the optimized CPs is presented.

### 3.1. The Distribution Measurement of CPs

Zhu [22] indicated that the probability of correct matching and information content enjoy strong correlation. This means that the probability of correct matching increases with increasing information entropy. The information entropy of every CP can be obtained by defining the second order differential invariants as the descriptor. The second order differential invariants ***v*** are [23]:

(7) $$v=\left[\begin{array}{c} L_{x}L_{x}+L_{y}L_{y}\\ L_{xx}L_{x}L_{x}+2L_{xy}L_{x}L_{y}+L_{yy}L_{y}L_{y}\\ L_{xx}+L_{yy}\\ L_{xx}L_{xx}+2L_{xy}L_{xy}+L_{yy}L_{yy} \end{array}\right]$$

where *L_x_* and *L_y_* are the convolution by the one-dimensional Gauss kernel, and *L_xy_* is the convolution by the two-dimensional Gauss kernel.

In order to calculate the probability of entropy, the descriptor ***v*** is partitioned into multi-dimensional vector space. The partitioning is dependent on the distance between descriptors which is measured by Mahalanobis distance. The distance between descriptors ***v***_1_ and ***v***_2_ is:

(8) $$D(v_{1},v_{2})={\left[{\left(v_{1}-v_{2}\right)}^{T}\Lambda^{-1}(v_{1}-v_{2})\right]}^{1/2}$$

where *Λ* is the covariance matrix. *Λ* can be SVD decomposed into *Λ*^−1^ = ***G***^T^***PG***, where ***P*** is a diagonal matrix and ***G*** is an orthogonal matrix. Then, ***v*** can be normalized by ***P***, which represents ***v***_norm_ = ***P***^1/2^***Gv***. After the normalization, *D*(***v***_1_, ***v***_2_) can be rewritten as:

(9) $$D(v_{1},v_{2})=\left\|P^{1/2}G(v_{1}-v_{2})\right\|$$

The descriptor ***v*** can be partitioned into many cells of equal size. Then the probability of each cell is used to calculate the entropy of ***v***:

(10) $$H(v)=-\sum_{i}p_{i}\log(p_{i})$$

where *p_i_* = *n**_v_***/*n* is the probability of ***v***. *n**_v_*** and *n* are the number of CPs in each cell and the total number of CPs, respectively.

The CP distribution can be regarded as the spatial dispersion of points, which can be measured by the normalized mean distance between CP and the distribution center of CPs. The distribution center of CPs is:

(11) $$(\bar{x},\bar{y})=\left(\frac{\sum_{i=1}^{n}w_{i}x_{i}}{\sum_{i=1}^{n}w_{i}},\frac{\sum_{i=1}^{n}w_{i}y_{i}}{\sum_{i=1}^{n}w_{i}}\right)$$

and DM is computed as:

(12) DM=([∑i=1n(xi−x¯Ix)2+∑i=1n(yi−y¯Iy)2]/n)12

where *I_x_* and *I_y_* are the row and column of the region. The parameter *w_i_* is the weight of the CP *i*, which is obtained by *H*(***v***) as Equation (10).

The bigger the DM is, the better the CPs distribution is. The smaller the DM is, the more concentrated the CPs is.

### 3.2. Piecewise Correction Strategy with Optimized CPs

According to the previous analysis, piecewise correction is indispensable for LA images, and the distribution quality of CPs is important to RMSE. Consequently, the image is gridded to determine the piecewise location and simultaneously control the overall CPs distribution. In each grid, DM is employed to constrain the CP quality. By using these procedures, the distribution uniformity of CPs is guaranteed from global to local.

**Figure 5:**
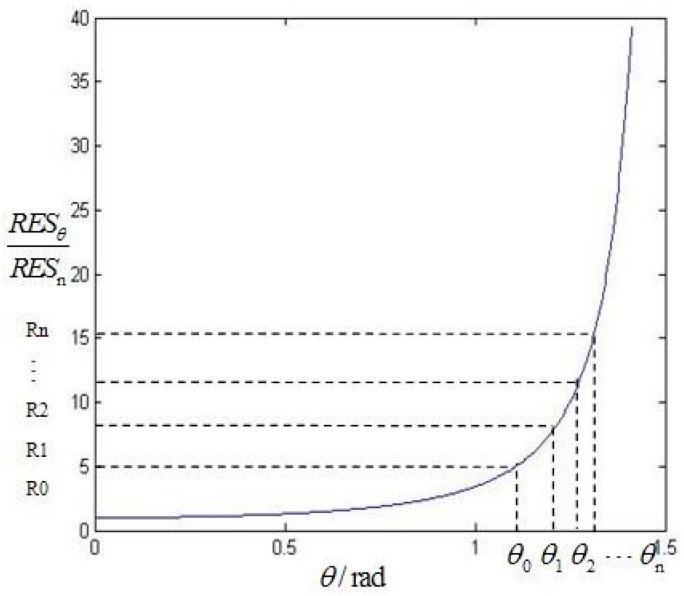
The gridded strategy in the *y* direction.

Since the resolution of LA images along the oblique direction (assuming the *y* direction) is nonlinear loss, the gridded strategies of the *x* direction and the *y* direction are different. For the *x* direction, the image is averagely divided into *M* subparts. For the *y* direction, the resolution variability is used to measure the distortion. Our aim is enable each grid to own approximately changing rate to the resolution. As a result, the gridded strategy of the *y* direction is shown as in Figure 5.

It can be seen that the larger the angle is, the smaller the division interval is. The gridded strategy for the *y* direction is:

(13) $$\theta_{n}=\arccos\sqrt{\frac{N}{\left(\frac{n}{\cos^{2}\theta_{\mathrm{top}}}+\frac{N-n}{\cos^{2}\theta_{\mathrm{bottom}}}\right)}}\;(0\leq n\leq N)$$

where *θ*_0_ and *θ_N_* are boundaries of the viewing angles. *N* is the subpart amount in the *y* direction*.*

**Figure 6:**
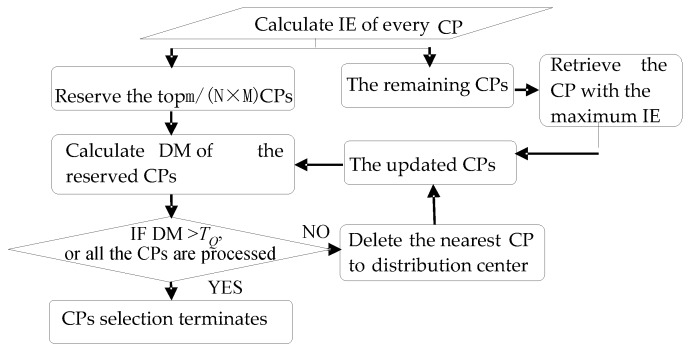
The CPs selection process in one gird.

**Figure 7:**
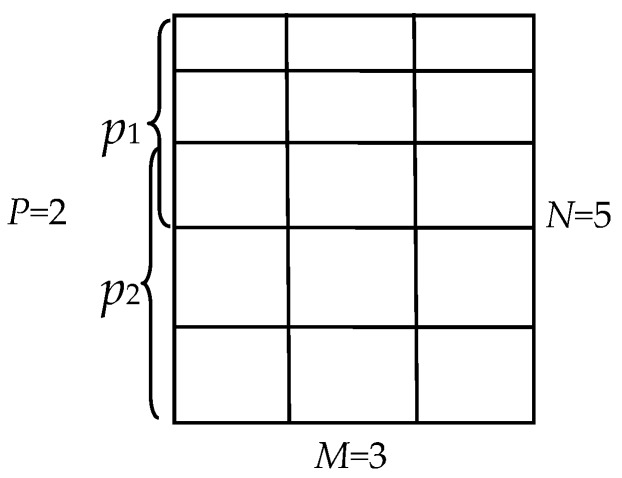
The grid division and the piecewise strategy (e.g., *p* = 2, *N* = 5, *M* = 3).

The detail of the proposed piecewise correction algorithm goes as follows:

1. Divide the whole image into *N* × *M* grids;
2. Set the maximum number of CPs needed to be selected in each grid. The maximum number is represented as *m*/(*N* × *M*), where *m* is the CPs total in the whole image. The following steps [(a)–(c)] should be executed in each grid:
  (a) Calculate the information entropy (IE) of every CP and put them in descending order, reserve the top *m*/(*N* × *M*) CPs, go to (b)). The remaining CPs form a spare set;
  (b) Calculate DM of the reserved CPs, if DM > *T_Q_*, or all the reserved CPs are processed, go to (c); If DM < *T_Q_*, delete the CP which is the nearest to the distribution center, retrieve the CP whose IE is the maximum value in the spare set, and re-execute (b); Here, *T_Q_* is the threshold.
  (c) Process all the grids. CPs selection terminates.
  Figure 6 illustrates the CPs selection process in one grid.
3. Partition the image into *P* correction subparts by merging adjacent grids along the *y* direction. To ensure the correction consistency at the piecewise edge, the adjacent subparts possess an overlap grid as shown in Figure 7.
4. Estimate the model parameters by using the selected CPs for each subpart. All the corrected subparts are integrated to obtain the final result.

## 4. Experimental Results and Analysis

**Figure 8:**
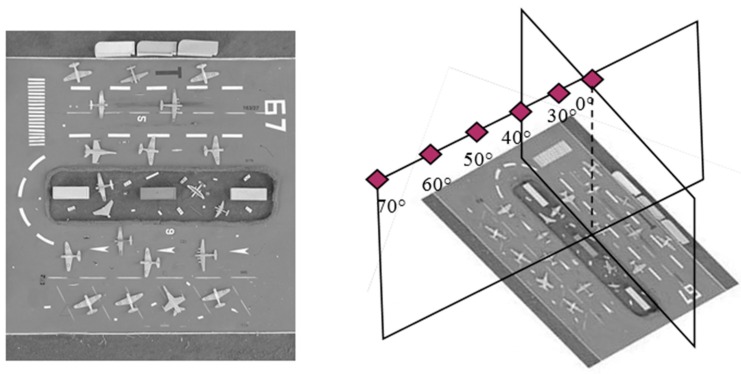
Images taken from semi-physical platform (the left is the reference image).

In order to comprehensively illustrate the performance of the proposed method, two groups of datasets are employed. One group is taken from our semi-physical platform by imaging nadir images for the reference image and off-nadir images for distortion images. The viewing angles are from 30° to 70° for an interval of 10° as shown in Figure 8.

**Figure 9:**
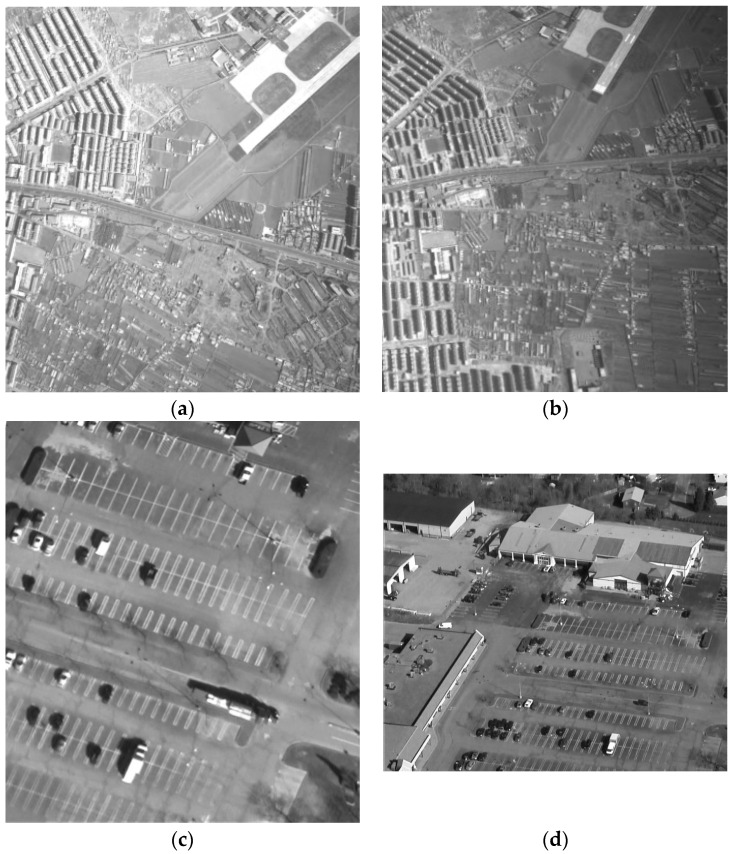
Two sets of aerial images (**a**) Reference image 1; (**b**) Distorted image 1; (**c**) Reference image 2; (**d**) Distorted image 2.

Another is aerial remote sensing images as shown in Figure 9, and their details are listed in Table 1. Our programs are implemented with MATLAB 2012a and the experiments are conducted on a Windows 7 computer with a Xeon 2.66 GHz CPU, and 8 GB memory.

### 4.1. Performance of CPs Extraction Based on Multi-View Simulation

This experiment is to examine the adaptability of the CPs extraction based on MVS for LA images. MVS of the reference image takes into account all distortions with enough accuracy which are caused by the variation of the imaging sensor optical axis. Therefore, the MVS is applied with very large parameter sampling ranges and small sampling steps. With the increase of the latitude *θ*, the distortions are increasingly larger. Especially near 90°, a little change of latitude *θ* can give rise to severe distortion. As a result, the sampling density would grow. Setting $u=\sqrt{2}$ satisfies this requirement. Similarly, since the image distortion in the high latitude is larger than that in the low latitude, the sampling step of the longitude *φ* should be reduced with the increasing of the latitude *θ*. We set Δϕ = 72°/*t*_k1_. The corresponding sampling parameters for MVS are demonstrated in Table 2. In our experiment, we set *m* = 4 and *n* = 2.

Table 3 lists the correct matching number which is conducted by ASIFT, SIFT, Harris-Affine [24], Hessian-Affine [7] and the proposed algorithm respectively. The computation costs of ASIFT, SIFT and the proposed algorithm are shown in Table 4.

It can be inferred that the performances of all the algorithms are deduced with the increasing of the imaging angle. The ASIFT and the proposed algorithm which can obtain more CPs than the other algorithms are more robust for LA images. For the distorted image with 70° as example, SIFT gets 0 CPs, the proposed algorithm gets 52 CPs, and the ASIFT gets 120 CPs. Compared with SIFT algorithm, the proposed algorithm successfully achieves the LA image correction, so it is worth spending a little bit more time. However, the ASIFT is time consuming compared with the proposed algorithm. The computation cost of ASIFT is nearly twice that of the proposed algorithm. Multiple matching of ASIFT results in the matching point redundancy which demands the huge calculating quantity and long computing time in the following process. This is indicative of the fact that the proposed algorithm is indispensable and more efficient than the other algorithms, especially for LA images. In addition, for the proposed algorithm, the situation whereby the result of 40° is more than the 30° one is related the sampling parameters *u*.

### 4.2. Performance of the Piecewise Correction with Optimized CPs

**Figure 10:**
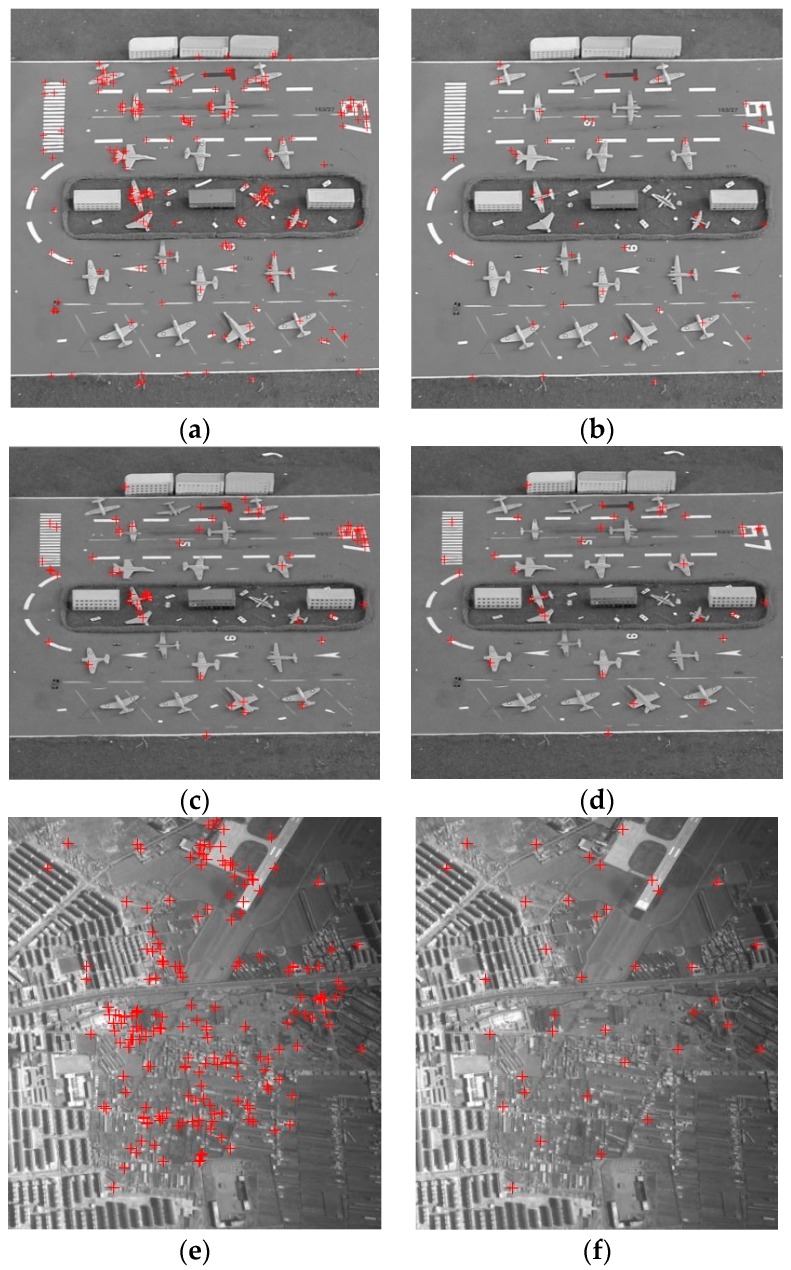

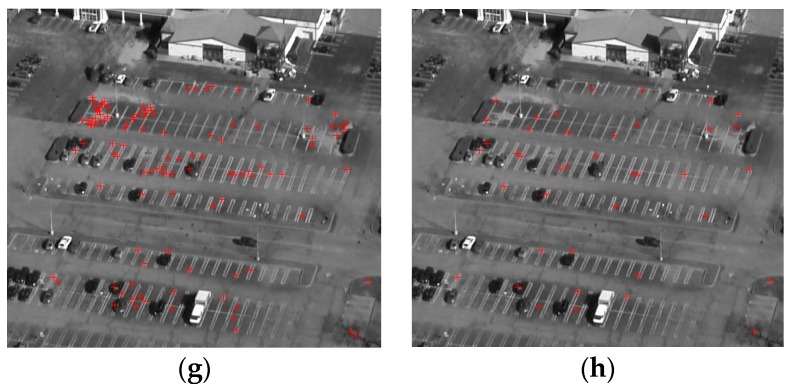
CPs optimization results (**a**) CPs distribution of 40° before optimization; (**b**) CPs distribution of 40° after optimization; (**c**) CPs distribution of 60° before optimization; (**d**) CPs distribution of 60° after optimization; (**e**) CPs distribution of aerial 1 before optimization; (**f**) CPs distribution of aerial 1 after optimization; (**g**) CPs of aerial 2 before optimization; (**h**) CPs of aerial 2 after optimization.

The total CPs of the whole image is adjustable. The number normally is 30~100. We set the total number *m* = 45. Considering the CPs number and computational complexity, we set *M* = 3, *N* = 5 and *p* = 2, just as shown in Figure 7. If *I_x_* equals to *I_y_*, the threshold *T_Q_* should be 0.25. Considering that *I_x_* and *I_y_* usually are different, *T_Q_* is adjusted to 0.35. For the CPs optimization process, the computation efficiencies of two CPs sets, which are obtained by the ASIFT and the proposed algorithm, are listed in Table 5. It is illustrated that the ASIFT is too time consuming to apply in practice. This is another proof that the proposed algorithm is indispensable. The CPs before and after optimization are shown in Figure 10.

It is obvious that the CPs gather around some objects before optimization. After the optimization, dense CPs are reduced. To examine the behavior of the proposed method, this part presents the correction results conducted by the total CPs from Section 2.2, the optimized CPs by using DM, the piecewise linear functions [15] by using the optimized CPs, and the piecewise strategy from Section 3.2, respectively. Except for the piecewise linear functions, the projective transformation is employed for the correction model. The test points in all the experiments have the same quantity and quality to ensure the equality of experiments, the number is set to 12. Here, RMSE is used to scale the correction accuracy. It can be given as:

(14) $$\mathrm{RMSE}=\sqrt{\sum_{i=1}^{n}\left(\Delta {x_{i}}^{2}+\Delta {y_{i}}^{2}\right)/n}$$

where *n* is the number of CPs, Δ*x_i_* and Δ*y_i_* are the CPs’ offset between the reference coordinates and the corrected coordinates. We also compute the correction errors of the *x* and *y* directions based on $\sqrt{\sum_{i=1}^{n}\Delta {x_{i}}^{2}/n}$ and $\sqrt{\sum_{i=1}^{n}\Delta {y_{i}}^{2}/n}$.

**Figure 11:**
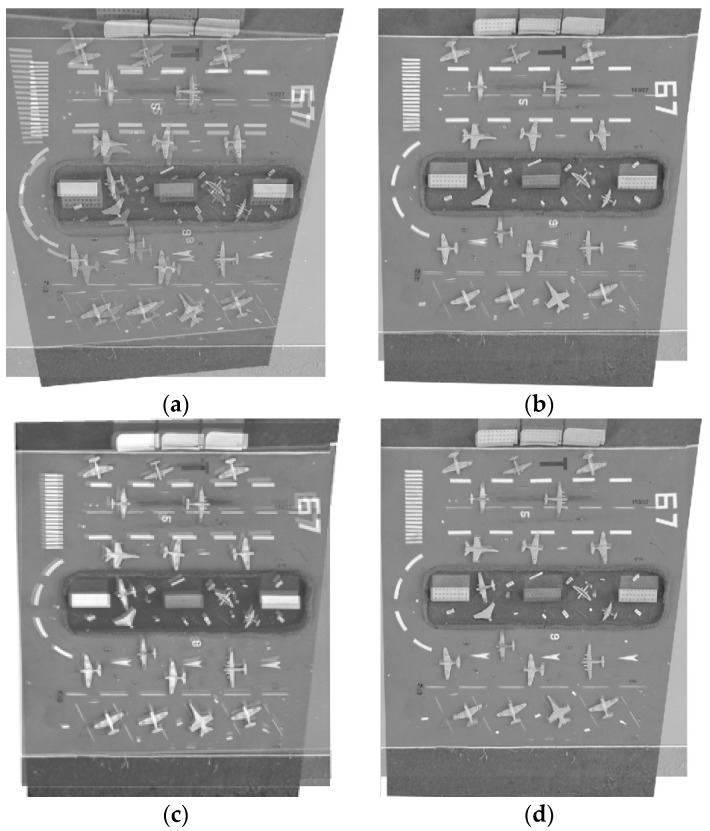
The correction results (for 60° as an example) (**a**) Correction results by the total CPs; (**b**) Correction results by the optimized CPs; (**c**) Correction results by the piecewise linear functions; (**d**) Correction results by the proposed piecewise strategy.

**Figure 12:**
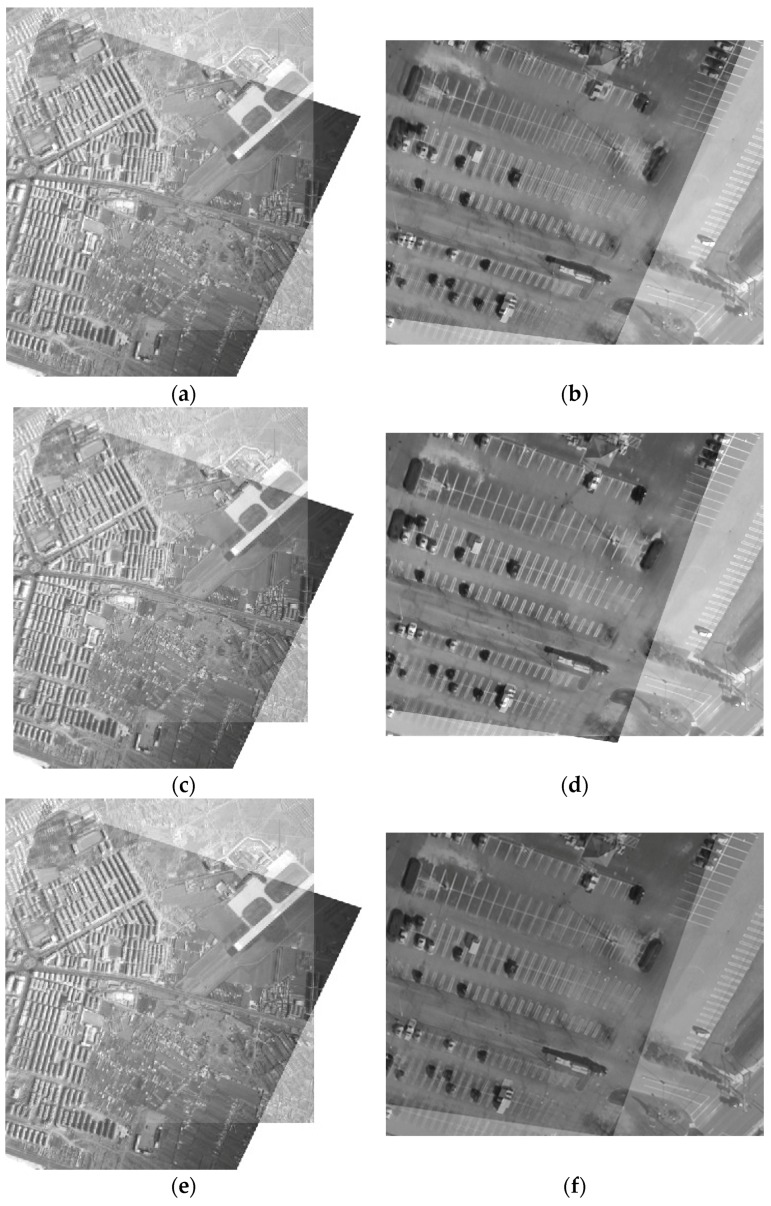

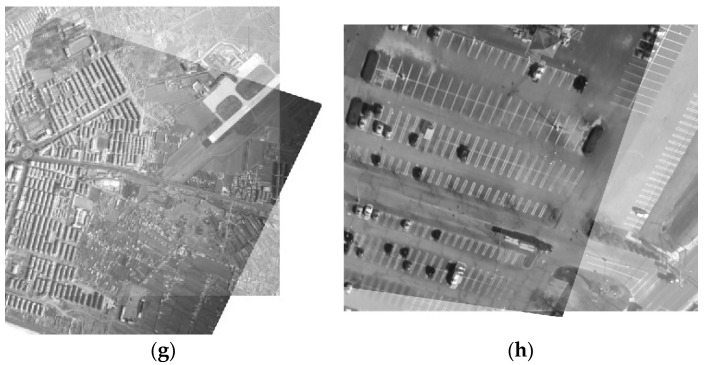
The correction results (**a**) The correction results by the total CPs of Aerial image 1; (**b**) The correction results by the total CPs of Aerial image 2; (**c**) The correction results by the optimized CPs of Aerial image 1; (**d**) The correction results by the optimized CPs of Aerial image 2; (**e**) The correction results by the piecewise linear functions of Aerial image 1; (**f**) The correction results by the piecewise linear functions of Aerial image 2; (**g**) The correction results by the proposed piecewise strategy of Aerial image 1; (**h**) The correction results by the proposed piecewise strategy of Aerial image 2.

Comparison of correction errors for the *x* and *y* direction is listed in Table 6, and the comparison of RMSEs is listed in Table 7.The mosaic results by different correction methods for 60° as an example are shown in Figure 11. Figure 12 shows the mosaic results of the aerial images 1 and 2 by different correction methods.

Visual inspection suggests that Figure 11a and the Figure 12b possess obvious double shadows. It means that correcting using the total CPs gives rise to a larger correction error. Similarly, Figure 11c and the Figure 12f also possess double shadows. This demonstrates the piecewise linear functions are not suitable for correcting LA images. A comparison of correction errors for the *x* and *y* direction is given in Table 6, and comparisons of RMSEs are listed in Table 7. It is observed that the optimized distribution of CPs is effective at improving the correction accuracy. After CP optimization, the reduction of the number of CPs doesn’t lead to a reduction of the correction accuracy, instead, the correction accuracy in the *x* and *y* direction is advanced, and the final RMSE is also improved. For the LA image with 60°, RMSE increases 12.03 pixels.

Compared with the model estimation based on the whole image, the proposed piecewise strategy improves the correction accuracy in both the *x* and *y* direction, and the final RMSE accordingly increases. The improvement of the correction accuracy increases with the increasing of the oblique angle especially for the *y* direction. For the LA image with 70°, RMSE of the proposed piecewise correction advances 0.45 pixels compared to the whole image correction. We can conclude that the proposed algorithm is effective and adaptable to correct LA distorted images.

## 5. Conclusions

Traditional correction methods are unsuitable to effectively correct remote sensing images acquired under seriously oblique conditions. On the basis of analyzing the characteristic of LA images, a new correction method is advanced. The proposed method simulates the multi-views of the reference image to ensure the effective matching of feature points, and uses the piecewise strategy based on optimized CPs to achieve highly accurate correction. The new method is analyzed theoretically and assessed experimentally by performing correction for LA images from our semi-physical platform and aerial remote sensing. Compared with the traditional methods, the new method can effectively correct LA images, and the correction accuracy is significantly improved. This paper has important value applied in geometric correction, especially under LA imaging conditions.

## Figures and Tables

**Table 1:** Introduction of the aerial remote sensing images.

Index	Size (pixel)	Resolution (m)	View Angle (°)	Rotation (°)	Location
image1	500 × 500	0.5	40	15	Liaoning, China
image2	800 × 700	0.1	60	15	Rhodes Island, USA

**Table 2:** The corresponding sampling parameters for multi-view simulation.

Factor *m*	1	2	3	4	5
θ	45°	60°	69°	75°	80°
Δϕ	48°	36°	24°	18°	12°

**Table 3:** Correct Matches of different algorithms (Number).

Index	ASIFT	SIFT	HarAff	HesAff	Proposed Method
30°	1126	155	38	33	212
40°	1733	123	31	19	225
50°	918	63	18	11	181
60°	685	18	9	5	96
70°	120	0	0	0	52
Aerial image 1	1247	112	29	17	215
Aerial image 2	128	41	10	7	137

**Table 4:** Computation Cost (Second).

Index	ASIFT	SIFT	Proposed Method
30°	11.25	4.58	6.11
40°	10.66	4.75	6.36
50°	9.76	4.07	5.89
60°	9.56	3.01	5.76
70°	9.56	2.64	4.18
Aerial image 1	9.27	4.66	6.77
Aerial image 2	10.86	4.85	6.14

**Table 5:** Computation Cost of CPs Optimization (Second).

Index	CPs by ASIFT	CPs by the Proposed Method
30°	30.83	5.74
40°	38.34	5.26
50°	29.76	3.21
60°	20.92	2.99
70°	3.56	1.68
Aerial image 1	36.88	4.84
Aerial image 2	3.69	3.56

**Table 6:** Comparison of correction errors for the *x* and *y* direction (pixel).

*Correction Errors*								
Index	Corrected by the Total CPs		Corrected by the Optimized CPs		The Piecewise Linear Functions		The Piecewise Correction	
	*x*	*y*	*x*	*y*	*x*	*y*	*x*	*y*
30°	0.73	1.11	0.69	0.93	0.87	1.19	0.67	0.93
40°	0.86	0.96	0.86	0.93	1.01	2.19	0.81	0.93
50°	1.05	2.46	0.89	1.76	1.51	3.12	0.83	1.57
60°	12.86	6.87	0.99	2.34	3.03	4.31	1.05	1.70
70°	19.65	9.04	1.59	3.74	5.27	7.10	1.51	2.92
Aerial 1	0.83	1.51	0.81	1.32	0.98	1.42	0.69	1.23
Aerial 2	6.98	14.38	1.96	3.12	4.66	6.34	1.84	2.66

**Table 7:** Comparison of *RMSE* (pixel).

*RMSE*				
Index	Corrected by the Total CPs	Corrected by the Optimized CPs	The Piecewise Linear Functions	The Piecewise Correction
30°	1.44	1.17	1.47	1.16
40°	1.29	1.27	2.21	1.24
50°	2.65	1.98	3.43	1.79
60°	14.57	2.54	5.26	2.07
70°	23.09	4.07	8.77	3.32
Aerial 1	1.72	1.58	1.76	1.42
Aerial 2	15.98	3.68	7.85	3.23
